# Nonsteroidal Anti-inflammatory Drugs (NSAIDs) and Collision Sports: The Need for Further Education in Rugby

**DOI:** 10.7759/cureus.108322

**Published:** 2026-05-05

**Authors:** Thomas J Papadimos, Brent Altenhof, Ravindera Uberoi, Justin P Reinert

**Affiliations:** 1 Anesthesiology, Surgery, and Critical Care, The University of Toledo College of Medicine and Life Sciences, Toledo, USA; 2 Anesthesiology, The University of Toledo College of Medicine and Life Sciences, Toledo, USA; 3 Pharmacy Practice, University of Toledo College of Pharmacy and Pharmaceutical Sciences, Toledo, USA

**Keywords:** anti-inflammatory drug, contact sport, return-to-sport, rugby, sport injury

## Abstract

Rugby is a collision sport and the fastest growing sport in the United States. Nonsteroidal anti-inflammatory drugs (NSAIDs), which can be acquired without prescription, are used by millions of individuals daily. Athletes in collision sports such as rugby overuse them, and the management of these analgesic anti-inflammatory drugs is gaining importance to healthcare providers in sports because of their potential organ toxicities. Athletes, amateur and elite, may misconstrue the perceived advantages of NSAIDs and seem to be unaware of the serious complications arising from their overuse. This overuse of NSAIDs by athletes should cause heightened health concerns to players, coaches, and physicians when athletes are administered or self-administer these drugs. Key educational messages include avoidance of prophylactic and prolonged unsupervised use of NSAIDs, understand the risks of analgesic masking, make proper return-to-play decisions, and related organ toxicities. More education and awareness regarding NSAIDs and their side effects are needed.

## Editorial

Rugby is truly a collision sport with potentially serious consequences, let alone daily match and practice injuries [1,2]. Pain and injury are common. It is one of the fastest growing sports in the United States [3] and is played in over 120 countries by over eight million individuals [4,5].

Recently, a review of worldwide rugby injuries analyzed by exposure type, anatomic location, injury type, rugby format, injury outcome, and sex involving nearly 150 reports, over 8,000,000 player hours, and over 95,000 rugby injuries found an injury incidence of 71.6 per 1000 player hours [6]. It is a fast, high-impact sport that results in a variety of injuries, some serious [3]. Rugby is played without the protective equipment of American football (there are more rules in rugby regarding what is appropriate tackling) [7]. It is a sport in which analgesic management and nonsteroidal anti-inflammatory drugs (NSAIDs), which are available over the counter, are acquiring increasing importance because of the potential organ toxicities that their use presents (in fact, over 30 million people consume NSAIDS globally on a daily basis) [8,9]. Athletes’ overuse of these over-the-counter analgesics has become a concern in elite and adolescent athletes [8,10]. While NSAID use is prevalent, the associated risk to athletes is not well understood [11]. The complications of NSAID use include gastrointestinal, cardiovascular, hepatic, renal, and neural effects [8,11-15].

It must be noted that many athletes believe that there is an ergogenic effect of NSAID use before and after exercise/match play. Extensive work by Holgado, Cornu, de Oliveira, and Pham have demonstrated that this belief is not supported by the evidence [16-19]. In fact, these authors indicate that NSAIDs are ergolytic, and this seems to occur through a negative effect on mitochondrial function [20]. Furthermore, NSAIDs introduce a clinically meaningful risk of analgesic masking. Pharmacologic suppression may permit premature return-to-play (RTP) and progression of subclinical pathology. Observational data demonstrate a high prevalence of NSAID use among elite athletes, including patterns of routine and prophylactic administration prior to competition, suggesting that utilization frequently extends beyond clearly defined therapeutic indications [21-24]. This is particularly concerning given that NSAIDs do not address the underlying biomechanical/structural injury, while only lessening the perceived pain although the ongoing injury persists. The implications of analgesic masking are amplified by repetitive, high-energy impacts. Athletes may unknowingly exacerbate an underlying injury, increasing the risk of injury progression, delayed recovery, or even reinjury. RTP decision-making in this context can be complex. The International Olympic Committee explicitly discourages prophylactic analgesic use for anticipated pain, emphasizing that pharmacologic therapy should be reserved for clearly defined, injury-based indications [25].

The NSAID mechanism of action involves blocking the metabolism of prostaglandins through the inhibition of cyclooxygenases (COX1 and COX 2) [8,13]. They assist in the creation of prostaglandin H2, a vital step in prostaglandin production, and through this blockade, they exert their anti-inflammatory effect. However, this mitigation of the effects of COX 1 is the main source of the toxicities encountered in NSAID use [13]. The NSAIDs also inhibit the formation of leukotrienes, which are also important in the anti-inflammatory process [8].

Complications from NSAID overuse are a serious concern in sports. Rugby players with a history of gastrointestinal problems, higher use dosages of NSAIDs, and an extended duration of use, are at risk of bleeding, nausea, vomiting, and abdominal discomfort [8]. Rugby training, in and of itself, has been demonstrated to cause intestinal endothelial damage and permeability as compared to the resting state of the athlete, thereby creating an environment for complications when NSAIDs are used frequently by athletes [26]. Chantler et al. specifically examined rugby training on gut permeability markers and demonstrated “increased intestinal endothelial cell damage and permeability compared to rest" [26]. Tso et al. demonstrated weight gain, increased systolic blood pressure, increased pulse wave velocity, and increased mitral annular velocities in early diastole [27]. They reported that excessive NSAID use in adolescence was associated with an increased cardiovascular risk, as did the work of Hall et al. [27,28]. NSAIDs are among the primary pharmaceutical causes of liver damage [29]. Add into the equation the collisions in rugby where abdominal trauma and dehydration are frequently encountered, hepatic complications can become a reality. Excessive use of NSAIDs may cause confusion, vision problems, drowsiness, headache, tinnitus, diplopia, and strokes [30,31]. It is quite evident that these side effects may exacerbate the potential for collisions in rugby and their consequences. Damage to the kidneys is another serious side effect of NSAIDs. The renal system receives a quarter of the cardiac output, and this makes the kidneys very vulnerable to NSAID-induced toxicity [32]. The damage to the kidney is twofold: inflammatory damage and functional damage [33]. Many players suffer flank injuries, and this lends itself to potentiating injury to the renal system. Although not necessarily related to practice or gameday encounters, prolonged use of NSAIDs may affect the regulation of reproductive function by reducing sperm count, sperm motility, and damage to seminiferous tubules, especially long-term use [34-36].

The overuse of NSAIDs should cause a heightened concern to players, coaches, physicians, and other support staff when players are administered or are self-administering these drugs [10,21,27,37-39]. Furthermore, rugby is a sport that can leave those retired from the game with an increased level of joint pain and prescription pain medication use than their non-rugby playing peers, thereby leading to the possibility of lifelong, or at least an extended use of NSAIDs [40]. Key educational messages for athletes and the staff that supports them include: avoidance of prophylactic and unsupervised prolonged use of NSAIDs, understanding the risks of analgesic masking, awareness of dehydration-related risks, as well as the gastrointestinal, cardiac, neural, and renal risks of NSAID administration. More research on prescribing practices, self-administration of NSAIDs, and pharmaceutical education in collision sports is warranted.
